# Karyotypic complexity rather than chromosome 8 abnormalities aggravates the outcome of chronic lymphocytic leukemia patients with *TP53* aberrations

**DOI:** 10.18632/oncotarget.13106

**Published:** 2016-11-04

**Authors:** Gonzalo Blanco, Anna Puiggros, Panagiotis Baliakas, Anastasia Athanasiadou, MªDolores García-Malo, Rosa Collado, Aliki Xochelli, María Rodríguez-Rivera, Margarita Ortega, Mª José Calasanz, Elisa Luño, MªTeresa Vargas, Javier Grau, Carolina Martínez-Laperche, Alberto Valiente, José Cervera, Achilles Anagnostopoulos, Eva Gimeno, Eugènia Abella, Evangelia Stalika, Jesús Mª Hernández-Rivas, Francisco José Ortuño, Diego Robles, Ana Ferrer, David Ivars, Marcos González, Francesc Bosch, Pau Abrisqueta, Kostas Stamatopoulos, Blanca Espinet

**Affiliations:** ^1^ Laboratori de Citogenètica Molecular, Laboratori de Citologia Hematològica, Servei de Patologia, Hospital del Mar, Barcelona, Spain; ^2^ Grup de Recerca Translacional en Neoplàsies Hematològiques, Cancer Research Programme, IMIM-Hospital del Mar, Barcelona, Spain; ^3^ Department of Experimental and Health Sciences, Universitat Pompeu Fabra, Barcelona, Spain; ^4^ Department of Immunology, Genetics and Pathology, Science for Life Laboratory, Uppsala University, Uppsala, Sweden; ^5^ Hematology Department and HCT Unit, G. Papanicolaou Hospital, Thessaloniki, Greece; ^6^ Servicio de Hematología, Hospital Universitario Morales Meseguer, Murcia, Spain; ^7^ Servicio de Hematología, Consorcio Hospital General Universitario, Valencia, Spain; ^8^ Institute of Applied Biosciences, CERTH, Thessaloniki, Greece; ^9^ Laboratorio de Citogenética y Servicio de Hematología, Hospital Vall d'Hebron, Barcelona, Spain; ^10^ Servicio de Citogenética, Departamento de Genética, Universidad de Navarra, Pamplona, Spain; ^11^ Servicio de Hematología, Hospital Universitario Central de Asturias, Oviedo, Spain; ^12^ UGC de Hematología, Hospital Universitario Virgen del Rocío, Instituto de Biomedicina de Sevilla (IBIS), Sevilla, Spain; ^13^ Servei d'Hematologia, ICO-Hospital Germans Trias i Pujol, Institut de Recerca Contra la Leucèmia Josep Carreras (IJC), Universitat Autònoma de Barcelona, Badalona, Spain; ^14^ Laboratorio de Genética Hematológica, Servicio de Hematología, Hospital G.U. Gregorio Marañón, Instituto de Investigación Sanitaria Gregorio Marañón, Madrid, Spain; ^15^ Servicios de Genética y Hematología, Complejo Hospitalario de Navarra, Pamplona, Spain; ^16^ Unidad de Genética, Hospital Universitario La Fe, Valencia, Spain; ^17^ Servei d'Hematologia, Hospital del Mar, Barcelona, Spain; ^18^ Servicio de Hematología, Hospital Universitario de Salamanca, IBSAL, IBMCC, Centro de Investigación del Cáncer, Universidad de Salamanca, CSIC, Salamanca, Spain; ^19^ Servicio de Hematología, Hospital Txagorritxu, Vitoria, Spain

**Keywords:** CLL, TP53 aberrations, chromosome 8 abnormalities, complex karyotype, prognosis

## Abstract

Patients with chronic lymphocytic leukemia (CLL) harboring *TP53* aberrations (*TP53*abs; chromosome 17p deletion and/or *TP53* mutation) exhibit an unfavorable clinical outcome. Chromosome 8 abnormalities, namely losses of 8p (8p−) and gains of 8q (8q+) have been suggested to aggravate the outcome of patients with *TP53*abs. However, the reported series were small, thus hindering definitive conclusions. To gain insight into this issue, we assessed a series of 101 CLL patients harboring *TP53* disruption. The frequency of 8p− and 8q+ was 14.7% and 17.8% respectively. Both were associated with a significantly (*P* < 0.05) higher incidence of a complex karyotype (CK, ≥3 abnormalities) detected by chromosome banding analysis (CBA) compared to cases with normal 8p (N-8p) and 8q (N-8q), respectively. In univariate analysis for 10-year overall survival (OS), 8p− (*P* = 0.002), 8q+ (*P* = 0.012) and CK (*P* = 0.009) were associated with shorter OS. However, in multivariate analysis only CK (HR = 2.47, *P* = 0.027) maintained independent significance, being associated with a dismal outcome regardless of chromosome 8 abnormalities. In conclusion, our results highlight the association of chromosome 8 abnormalities with CK amongst CLL patients with *TP53*abs, while also revealing that CK can further aggravate the prognosis of this aggressive subgroup.

## INTRODUCTION

The great majority (~80%) of patients with chronic lymphocytic leukemia (CLL) carry one of the chromosomal aberrations routinely analyzed by fluorescence *in situ* hybridization (FISH), namely deletions of 13q [del(13q)], 11q [del(11q)] and 17p [del(17p)] as well as trisomy 12. These aberrations are associated with distinct clinical outcome [1, 2]. In particular, del(17p) cases have the worst clinical outcome and the shortest overall survival (OS). Of note, it has been described that the remaining *TP53* allele in del(17p) cases is generally mutated, although some CLL patients may harbor isolated *TP53* mutations [3]. Taking into account both *TP53* deletions and mutations, the rate of *TP53* aberrant CLL cases peaks at 10% at diagnosis [4, 5] and may even exceed 40% at disease progression, mainly in patients previously treated with chemotherapy [6]. In addition, genomic complexity detected by chromosome banding analysis (CBA) or genomic microarrays also predicting rapid disease progression, is correlated to *TP53* disruption in a significant proportion of patients [7–9].

*TP53* aberrations (*TP53*abs, namely del(17p) and/or *TP53* mutations) are associated with low response rates to standard chemoimmunotherapy [10]. Newly introduced signaling inhibitors represent a major advance for this group of patients, showing an improved overall response and progression-free survival even in refractory CLL cases [11–13].

Nonetheless, some CLL cases with *TP53*abs remain asymptomatic for extended periods of time, pointing to multiple associated genetic factors underlying the heterogeneity of this group of patients [14, 15].

Alterations of chromosome 8, and particularly losses of 8p (8p−, affecting several regions, from 8p11 to 8p23) and gains of 8q (8q+, usually involving 8q24, where *MYC* is located), have been suggested as new prognostic markers in CLL, even for patients harboring *TP53*abs. Although initial microarray studies identified chromosome 8 alterations in around 5% of the general CLL population, subsequent analyses detected an increased frequency in patients with del(17p), ranging from 28 to 80%; 8p− and 8q+ alterations were frequently concomitant and displayed an independent correlation with a shorter OS [16–21].

However, due to the small size of the evaluated cohorts, definitive conclusions could not be drawn regarding the actual impact of chromosome 8 abnormalities in CLL patients harboring *TP53*abs. In the present study we sought to obtain more insight into this issue by analyzing a series of 101 CLL patients with *TP53*abs, especially focusing on the potential prognostic value of 8p losses and 8q gains in this poor prognostic group of patients.

## RESULTS

### Patients

A total of 101 patients carrying *TP53* abnormalities were included in this study: 92 cases with del(17p) and 9 additional cases with *TP53* mutations. Demographic, clinical and biological data for the entire cohort are summarized in Table 1.

**Table 1:** Baseline characteristics of patients at diagnosis and last follow-up

Patients characteristics (*n* =101)	
Age at diagnosis	64 (42–87)
Male	69 (68.3%)
Diagnosis	
MBL*	6 (5.9%)
CLL	95 (94.1%)
Binet stage** (*n* =90)	
A	57 (63.3%)
B	23 (25.6%)
C	10 (11.1%)
B-symptoms (*n* = 67)	6 (9%)
Adenopathies (*n* = 71)	37 (52.1%)
Splenomegaly (*n* =68)	12 (17.6%)
Hepatomegaly (*n* =68)	6 (8.8%)
Absolute white blood cell count (× 10^9^/L) (*n* = 71)	20 (3.8–372)
Absolute lymphocyte count (× 10^9^/L) (*n* =68)	15 (1–369)
Hemoglobin (g/dL) (*n* = 68)	13.8 (7–18)
Platelets (×10^9^/L) (*n* = 68)	196 (2–356)
Lactate dehydrogenase (IU/L) (*n* = 64)	335 (180–959)
Beta-2 microglobulin (mg/L) (*n* = 59)	2.4 (1–8.4)
Unmutated *IGHV* (*n* =32)	27 (84.4%)
Mutated *NOTCH1* (*n* = 19)	2 (10.5%)
**Last follow-up**	
Treated patients (*n* =97)	81 (83.5%)
Time to first treatment (months, 95% CI) (*n* = 96)	23 (14–33)
Deaths	66 (65.3%)
Overall survival (months, 95% CI) (*n* = 99)	88 (67–108)
Follow-up (months)	62 (0–201)

Values are given as median (range) or number (%). Hemoglobin is expressed as mean (range).

* Although 6 patients were diagnosed as MBL (monoclonal B-cell lymphocytosis), all of them had already progressed to CLL at the time of study.

** Some centers only provided information regarding the Binet stage without the physical examination and the analytical parameters.

### Chromosome 8 alterations

Overall, 23/101 cases (22.8%) displayed chromosome 8 alterations. In detail, 11/75 patients (14.7%) showed 8p−, while 18/101 cases (17.8%) had 8q+ with different alteration patterns (Table 2). In 6/75 patients (8%) both abnormalities were concomitant; baseline characteristics of patients with concomitant 8p− and 8q+ are shown in Supplementary Table S1. Four of the cases with concomitant 8p− and 8q+ also presented two altered clones with shared FISH patterns (Table 2).

**Table 2:** Chromosome banding analysis and FISH results in patients with alterations of chromosome 8

Chromosome Banding Analysis			FISH		
ID	Karyotype	% del(17p)	Chromosome 8 alteration		
			%	FISH patterns*	
1	46,XX,del(8)(p21),add(10)(q26),add(17)(p13),+2ac[5]/47,XX,+12,add(17)(p13),del(18)(q21),add(22)(q13)[3]	80	20	1O2G	8p-
2	−	95	75	1O2G	
3	46,XX,add(6)(q24),add(14)(q32.3),i(17)(q10)[6]/46,XX[8]	95	75	1O2G	
4	48,X,-X,-17,+4mar[13]/44,X,-X,del(6)(q23),-9,-13,-17,-21,+3mar[3]/46,XX[4]	86	50	1O2G	
5	45,XY,-5,-9,-15,add(17)(p13),+18,-21,+2mar[13]/46,XY[37]	70	17	1O2G	
6	44,X,-X,-6,der(13;15)(q10;q10),add(17)(p13),-20,+mar[13]/46,XX[7]	95	23/10	1O2G/1O3G	8p- and 8q+
7	46,X,der(X),add(8)(p23),del(13)(q12q22),add(17)(p13)[11]/46,XX[13]	10	32	1O3G	
8	−	95	64	1O3G	
9	45,XY,add(3)(q29),del(4)(q26q35),der(7)(1p36→1p32::7p22®7q32::15q22→15q26), -8,der(9),del(13)(q21q34),-15,-17,-18,+19, add(19)(p13),+2mar,+ac[17]/46,XY[3]	78	34/21	1O3G/2O3G	
10	−	95	66/31	1O3G/1O2G	
11	45,XY,add(6)(q22),del(11)(q11q22),-17[15]/44,XY,add(6)(q22),del(11)(q11q22),-17,-20,-22,+mar[2]	87	40/24	1O3G/1O2G	
12	46,XX,del(13)(q14q21)[2]/45,X,-X,del(13)(q14q21)[3]/45,XX,add(3)(q27),t(9;10)(q21;q22), +12,der(12)t(12;17)(q11;p11),del(13)(q14q21),-14,-17[7]/46,XX[8]	70	62	2O3G	8q+
13	46,XY[30]	14	88	2O3G	
14	46,XY[13]	80	82	2O3G	
15	47,XY,+12[8]/46,XY,add(1)(p34),add(2)(q34),t(11;22)(p14;q11),+12,-22[15]/46,XY[11]	75	18	2O3G	
16	45,XY,add(6)(p11),-22[13]/46,XY,i(17)(q10)[5]/46,XY[16]	16	57	2O3G	
17	45,XY,del(6)(q?),-9,add(14)(q32),-22,+mar[9]/46,XY,del(6)(q?),add(17)(p13),add(19)(q13)[21]	55	23	2O3G	
18	43,X,-X,del(2)(p15),+4,-7,add(11)(q21),-12,-13,add(14)(q32),add(17)(p11.2)[6]/46,XX[9]	19	14	2O3G	
19	−	70	66	2O4G	
20	−	90	81	2OnG	
21^#^	43-44,X,-Y,add(2)(q37),dic(3;11)(p21;q23),-8,add(8)(q24),add(16)(q24),-17,add(17)(p13), -18,+mar1,+mar2[cp18]/46,XY[11]	68	18	3G	
22^#^	45,XY,-15,add(17)(p13)[7]/45,XY,-15,add(17)(p13),add(21)(p13)[5]/ 44,XY,der(3)t(3;4)(p26;q21),-4,-15,add(17)(p13)[2]/46,XY[10]	53	15	3G	
23^#^	42,X,-Y,del(4)(p12p15),-8,add(8)(q24),-13,add(14)(p13),-15,-17,add(21)(p13),+mar[17]/46,XY[3]	38	77	3G	

* O: LPL (8p21) signal in orange, G: *MYC* (8q24) signal in green.

# Only the *MYC* probe was analyzed.

In 12/23 abnormal patients, chromosome 8 alterations were identified prior to treatment initiation either at diagnosis or within the following 12 months (*de novo*). In contrast, in the remaining 11 cases chromosome 8 analysis was performed later during the disease course (*n* = 10/11) or after treatment administration (*n* = 8/11) (median time to chromosome 8 analysis: 36 months, range: 9–132). In three of the latter cases, retrospective FISH analysis of stored diagnostic material disclosed the presence of chromosome 8 alterations without del(17p); in one of these three cases, a different clonal distribution of the abnormal chromosome 8 clones was observed between the two time points (Supplementary Figure S1). Altogether, chromosome 8 alterations could be considered *de novo* in 65.2% of cases (15/23). Unfortunately, no previous cytogenetic studies from the remaining patients (8/23) were available to elucidate the acquisition pattern of chromosome 8 and *TP53*abs.

For the purpose of this study, two types of comparisons were established: patients with 8p– *vs.* those with normal 8p (N-8p) and patients with 8q+ *vs.* those with normal 8q (N-8q).

### Other cytogenetic abnormalities

At the time of chromosome 8 analysis by FISH, 48.3% of patients presented del(13q), 12.9% trisomy 12 and 5.7% del(11q). Among the 66 patients with available CBA, 53 (80.3%) presented an abnormal karyotype and 31 (47%) showed a complex karyotype (CK).

When comparing cases with 8p− or 8q+ versus those with N-8p or N-8q, respectively, no differences regarding del(13q), trisomy 12 and del(11q) frequencies were detected. Both the 8p− and the 8q+ groups carried a higher median percentage of 17p-deleted cells compared to cases with normal chromosome 8, although differences were only significant for the 8p− *vs.* N-8p comparison (87% *vs.* 50%, *P* = 0.001; 70% *vs.* 48%, *P* = 0.07). As for CBA, both the 8p− and the 8q+ group displayed a higher median number of chromosomal alterations (7 *vs.* 3, *P* = 0.041 and 6.5 *vs.* 2, *P* = 0.001 respectively), therefore displaying a higher frequency of CK (100% *vs.* 45.9%, *P* = 0.006 and 85.7% *vs.* 36.5%, *P* = 0.002 respectively) compared to N-8p and N-8q cases.

### Demographic and baseline characteristics

When comparing 8p− *vs.* N-8p and 8q+ *vs*. N-8q cases the only statistically important differences concerned female prevalence (63.6% *vs.* 31.3%, *P* = 0.049) and lower hemoglobin levels (12.8 *vs.* 14 g/dL, *P* = 0.018) in the 8p− group.

### Therapy

Overall, with a median follow up of 62 months (0–201), 81/97 cases (83.5%) had received treatment. Of note, in 34/93 cases (36.6%), therapy was administered prior to chromosome 8 testing. In 17/23 cases with 8p− and/or 8q+ (73.9%), these alterations were observed before treatment, whereas in the remaining six patients therapy-related acquisition could not be formally excluded as no relevant sample from the time of diagnosis was available for testing.

Among treatment-naïve cases, both 8p− and 8q+ groups exhibited a shorter time to first treatment (TTFT; 11 *vs.* 45 months for 8p− *vs.* N-8p and 15 *vs.* 45 months for 8q+ *vs.* N-8q) although differences did not reach statistical significance.

### Survival analysis

**Figure 1:**
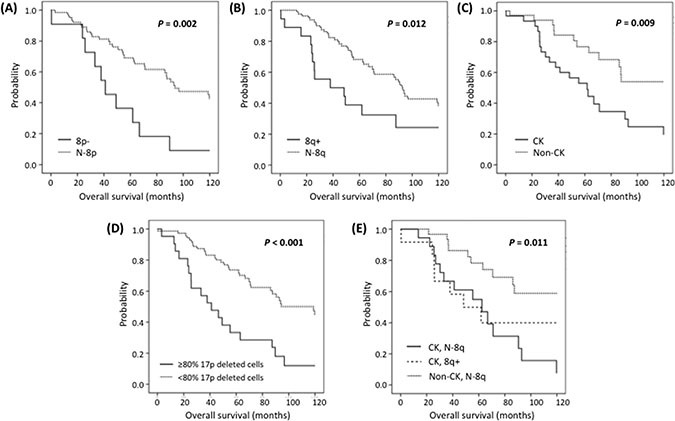
Kaplan-Meier plots for ten-year OS in patients carrying (A) 8p−, (B) 8q+, (C) CK, (D) ≥80% 17p deleted cells and (E) CK with 8q+ or N-8q

With a median follow-up of 62 months (range: 0–201), 35 patients (34.6%) remained alive. Ten-year survival analysis revealed a significant shorter median OS for both 8p− and 8q+ patients compared with N-8p and N-8q group respectively (41 *vs.* 94 months; *P* = 0.002 and 38 *vs.* 92 months; *P* = 0.012 respectively) (Figure 1, Table 3). Interestingly, even though the number of cases with concomitant 8p− and 8q+ was low (*n* = 6), they exhibited a significant shorter OS compared to patients with isolated chromosome 8 alterations (26 *vs.* 88 months, *P* = 0.016). An elevated percentage of 17p-deleted cells also was related to inferior outcome, with the optimal cut-off being ≥80% of deleted cells (*P <* 0.001).

**Table 3:** Univariate and multivariate analysis on ten-year overall survival

Variable		Univariate		Multivariate	
		Median OS (95% CI) in months	*P*-value	Hazard ratio (95% CI)	*P*-value
**Loss of 8p by FISH**	**8p−**	41 (24–58)	0.002	NA	NA
	**N-8p**	94 (60–127)			
**Gain of 8q by FISH**	**8q+**	38 (0–85)	0.012	1.23 (0.52–2.90)	0.642
	**N-8q**	92 (84–100)			
**Complex karyotype**	**Yes**	62 (40–83)	0.009	2.47 (1.11–5.49)	0.027
	**No**	NR			
**Percentage of cells with 17p deletion**	**≥ 80%**	41 (21–61)	< 0.001	2.21 (1.02–4.80)	0.046
	**< 80%**	94 (60–129)			

NA, not assessed; NR, not reached.

Regarding chromosomal abnormalities detected by CBA, patients with CK exhibited a shorter OS (*P* = 0.009), which was independent of the presence or absence of 8q+ (48 *vs.* 62 months, *P* = 0.538) (Figure 1). Similar results were obtained when cases analyzed after treatment were excluded from the statistical analysis (Supplementary Figure S2). On multivariate analysis including 8q+, ≥ 80% of 17p-deleted cells and CK only ≥ 80% of 17p-deleted cells (*P* = 0.046, HR = 2.2) and CK (*P* = 0.027, HR = 2.5) retained independent significance (Table 3). Multivariate analysis including 8p− cases could not be performed due to the low number of patients with available data from both CBA and 8p status.

## DISCUSSION

Mounting evidence suggests that within CLL patients with *TP53*abs additional genetic features may influence the clinical outcome [17, 22, 23]. More specifically, alterations in chromosome 8 are more frequently detected in CLL patients with *TP53*abs and have been proposed as potentially prognostic markers in this aggressive group. Nevertheless, large studies focusing on the characterization and clinical outcome of this subset of patients are lacking. In order to address this issue, we retrospectively analyzed 101 CLL patients with *TP53*abs from our collaborating institutions. The frequency of 8p− was 14.7%, whereas 8q+ was detected in 17.8% of patients, being concomitant in 30% of cases with chromosome 8 alterations. To the best of our knowledge, this is the largest cohort of patients with *TP53*abs confirming the enrichment of chromosome 8 abnormalities in this disease subgroup. The frequencies reported herein are lower than those in previous studies, which, however, concerned significantly smaller cohorts, thus raising the possibility of selection bias [17, 19, 20].

The high percentage of *de novo* chromosome 8 alterations reported here suggests that 8p− and 8q+ can occur early in the course of the disease. This is in agreement with earlier reports of 8p− and 8q+ in treatment-naïve patients [20, 21]. Indeed, subsequent whole-exome sequencing studies analyzing clonal heterogeneity also suggested 8p− and 8q+ as early events during CLL development, whereas *TP53* aberrations seem to appear later in the course of the disease [24].

Regarding the clinical characteristics of patients with alterations in chromosome 8 at diagnosis, no significant differences could be identified between groups except for gender distribution and hemoglobin levels. As for the genetic characteristics, we found a higher percentage of 17p-deleted nuclei in patients with 8p− or 8q+. Furthermore, in accordance with previous reports, a higher rate of CK was associated with both chromosome 8 abnormalities (8p− and 8q+) [25, 26]. Concerning clinical outcome, even though both 8p− and 8q+ exhibited a shorter TTFT, differences did not reach statistical significance. Nonetheless, a decreased OS was observed for both 8p− and 8q+, with concomitant 8p− and 8q+ cases exhibiting the worst outcome. Concerning patients with isolated 8p− or 8q+, given the small number of cases, we could not statistically assess their potential impact on survival. Our findings indicate that co-existence of 8p− and 8q+ may have a potential clinical impact in CLL and should be better evaluated in future research. However, further studies in larger patient cohorts are needed to discern the clinical consequences of isolated 8p− or 8q+.

Karyotypic complexity has been reported to be significantly associated with *TP53*abs [7, 25, 27] and to have a negative impact on survival even within this aggressive subset of patients [8, 23, 27]. Our present study confirms and significantly extends previous reports highlighting the impact of genetic instability in the pathogenesis of the disease. Indeed, although 89% of cases with 8p− and/or 8q+ showed a CK, in the multivariate analysis only karyotypic complexity and the percentage of 17p-deleted nuclei maintained the statistical significance. This finding suggests that CK rather than chromosome 8 alterations is responsible for the dismal clinical evolution of these patients. Chromosome 8 alterations could reflect underlying genomic instability leading to karyotypic complexity and could suggest the implication of different genes such as *MYC* in 8q+ cases and tumor suppressors such as *TNFRSF10A/B* (8p21) in 8p− cases [20, 28]. Notably, however, almost 40% of cases with CK did not show 8p− or 8q+ but displayed the same poor outcome as those with chromosome 8 alterations and CK.

In conclusion, our results highlight the association of chromosome 8 abnormalities with CK amongst CLL patients with *TP53*abs, while also revealing that CK can further aggravate the prognosis of this aggressive subgroup. These findings further underscore the relevance of chromosome banding analysis, which has the potential to refine prognosis even amongst cases with altered *TP53*.

## MATERIALS AND METHODS

### Patient selection

For the purpose of this study, we evaluated CLL patients with *TP53*abs and available Carnoy fixed tumoral cells from 17 collaborating institutions in Spain and Greece (Supplementary Figure S3). All patients met the iwCLL diagnostic criteria [29]. A total of 101 patients carrying *TP53* abnormalities were identified: 92 cases with del(17p) detected by FISH and 9 additional cases with *TP53* mutations identified by Sanger sequencing. Of note, six patients had been diagnosed of CLL-like monoclonal B cell lymphocytosis (MBL) but had already progressed to CLL at the time of the study. Treatment initiation and response assessment followed standard criteria [29, 30]. The study was performed in accordance with national and international guidelines (Professional Code of Conduct, Declaration of Helsinki) and approved by the Ethics Committee of Hospital del Mar, Barcelona (2013/5093/I).

### Fluorescence *in situ* hybridization and chromosome banding analysis

Chromosome 8 alterations were assessed in the same Carnoy fixed tumoral cells (peripheral blood or bone marrow) which were used for the detection of *TP53*abs. Several samples were obtained from Parc de Salut MAR Biobank (MARBiobanc), Barcelona. The median time from diagnosis to study of chromosome 8 abnormalities was 15 months (range: 0–145) and 36.6% of the patients had received treatment prior to the analysis. Commercial FISH probes covering *LPL* (8p21) and *MYC* (8q24) genes (Abbott Molecular, Abbott Park, IL) were used in 75 and 101 patients, respectively. Moreover, data from routine FISH (D13S319, CEP12 and *ATM*) and CBA (72 h + TPA and/or CpG + IL2) were also available in 91 and 66 cases, respectively. Complex karyotypes (CK) were defined as the presence of three or more numerical/structural chromosomal abnormalities.

### Statistical analysis

Chi-square or Fisher exact tests were employed for discrete variables, while comparisons of continuous variables were assessed by the Mann–Whitney test. TTFT was defined as the time from diagnosis to the beginning of treatment and OS as the time from diagnosis to death or last follow-up. Both parameters were evaluated using Kaplan-Meier plots within the first ten years of follow-up. The effect of different covariates was assessed employing the log-rank test. Multivariate analysis according to the Cox proportional hazards regression model was used to evaluate the maintenance of their independent predictive value. Statistical analyses were performed using SPSS v.22 software (SPSS Inc, Chicago, IL, USA). *P-value*s below 0.05 were considered statistically significant. The optimal cut-off value for the percentage of cells harboring del(17p) was assessed using Maximally Selected Rank Statistics in R.

## SUPPLEMENTARY MATERIALS


