# Supplementary material for: Karyotypic complexity rather than chromosome 8 abnormalities aggravates the outcome of chronic lymphocytic leukemia patients with TP53 aberrations
Source: Oncotarget. 2016 Nov 4;7(49):80916–24. doi: 10.18632/oncotarget.13106 (PMC5348364; doi:10.18632/oncotarget.13106)
Supplement: Supplementary file 1 [file oncotarget-07-80916-s001.pdf]

Karyotypic complexity rather than chromosome 8 abnormalities aggravates the outcome of chronic lymphocytic leukemia patients with *TP53* aberrations

Supplementary Materials

Supplementary Table S1: Baseline characteristics at diagnosis of the six patients with concomitant presence of 8p– and 8q+

Patients characteristics (*n* = 6)	
Age at diagnosis	72 (51–76)
Male	3 (50%)
Diagnosis	
MBL	0
CLL	6 (100%)
Binet stage	
A	1 (16.7%)
B	4 (66.7%)
C	1 (16.7%)
B-symptoms	3 (50%)
Adenopathies	6 (100%)
Splenomegaly	2 (33.3%)
Hepatomegaly	1 (16.7%)
Absolute white blood cell count ($\times 10^9$/L)	34 (9.4–372)
Absolute lymphocyte count ($\times 10^9$/L)	27 (7.3–369)
Hemoglobin (g/dL)	12.7 (9–14)
Platelets ($\times 10^9$/L)	172.5 (34–223)
Lactate dehydrogenase (IU/L)	376.5 (214–522)
Beta-2 microglobulin (mg/L) (*n* = 4)	4 (2–8)

Values are given as median (range) or number (%). Hemoglobin is expressed as mean (range).

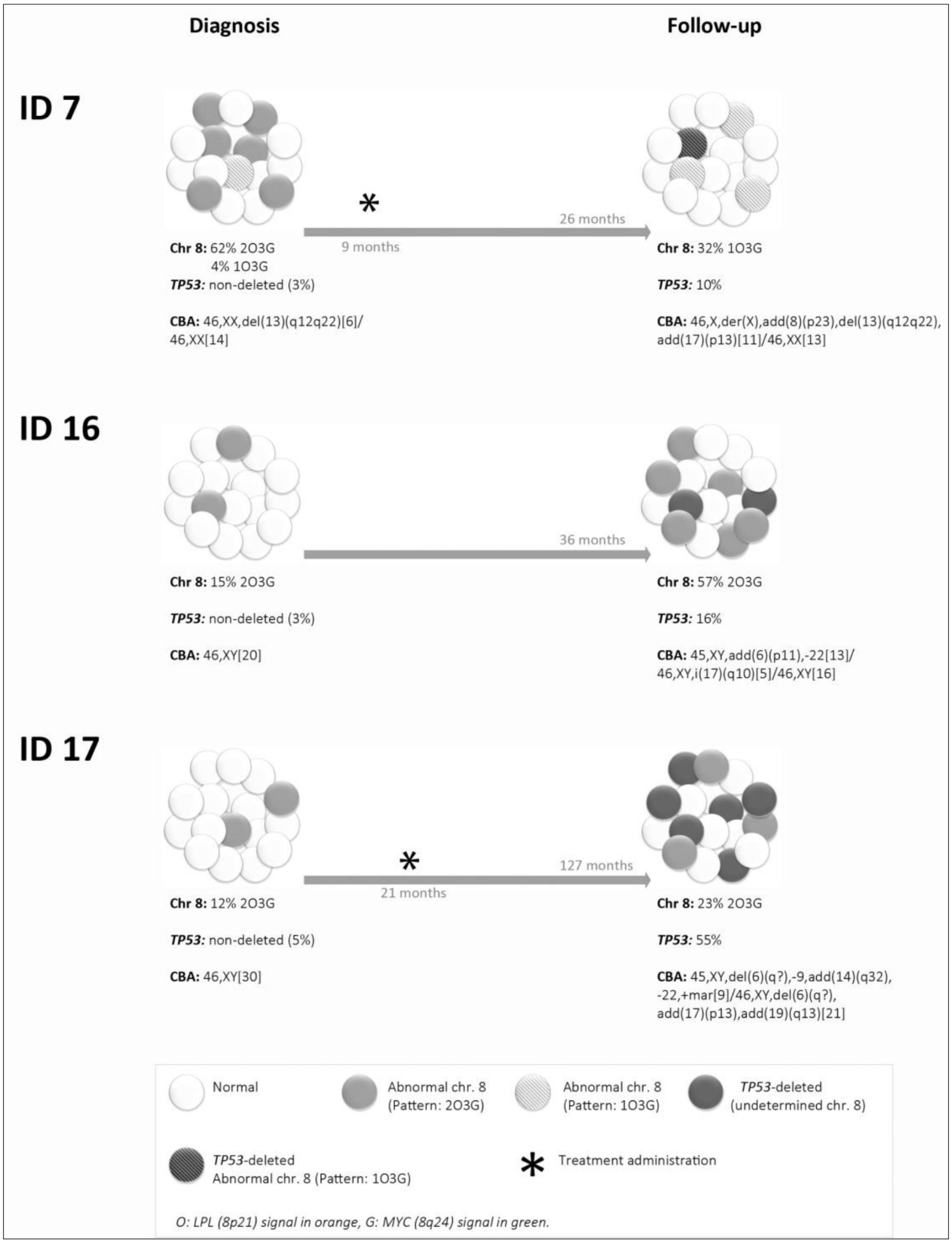

Supplementary Figure S1: Representation of the clonal evolution process in three patients with alterations of chromosome 8 at diagnosis and during the follow-up. Sequential analyses demonstrated the presence of chromosome 8 abnormalities at diagnosis and prior to the acquisition of *TP53* deletion. While in one case (ID 7) CBA from the second time-point showed concomitance of chromosome 8 and *TP53* abnormalities in the same tumoral clone, CBA results were not conclusive in the other cases (ID 16 and 17). As chromosome 8 analyses and *TP53* assessment by FISH were performed in different batches on interphase nuclei, the concomitance of both abnormalities could not be determined in these cases. Cut-off value for FISH positivity was established at ≥ 10%.

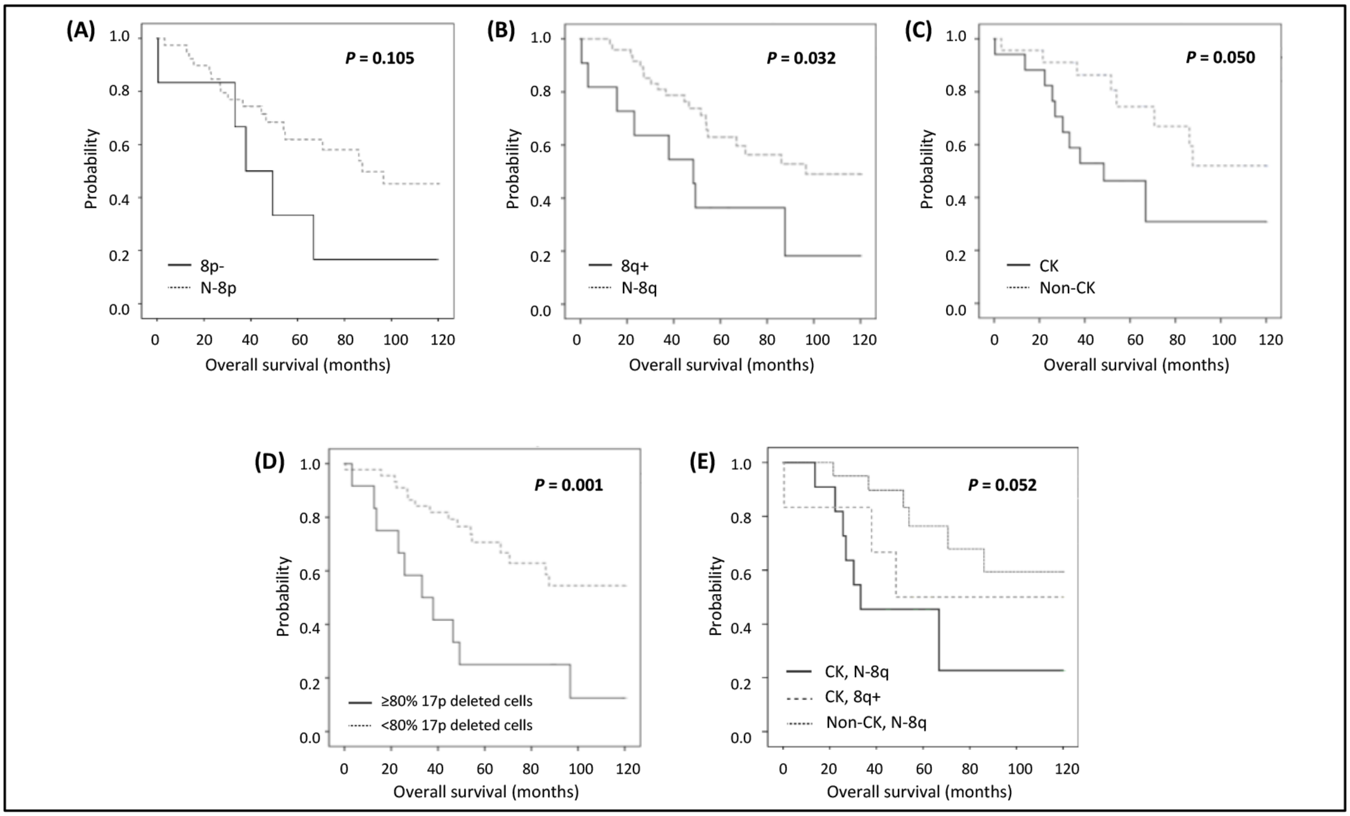

Supplementary Figure S2: Kaplan-Meier plots for ten-year OS in treatment-naïve patients carrying (A) 8p–, (B) 8q+, (C) CK, (D) ≥ 80% 17p deleted cells and (E) CK with 8q+ or N-8q.

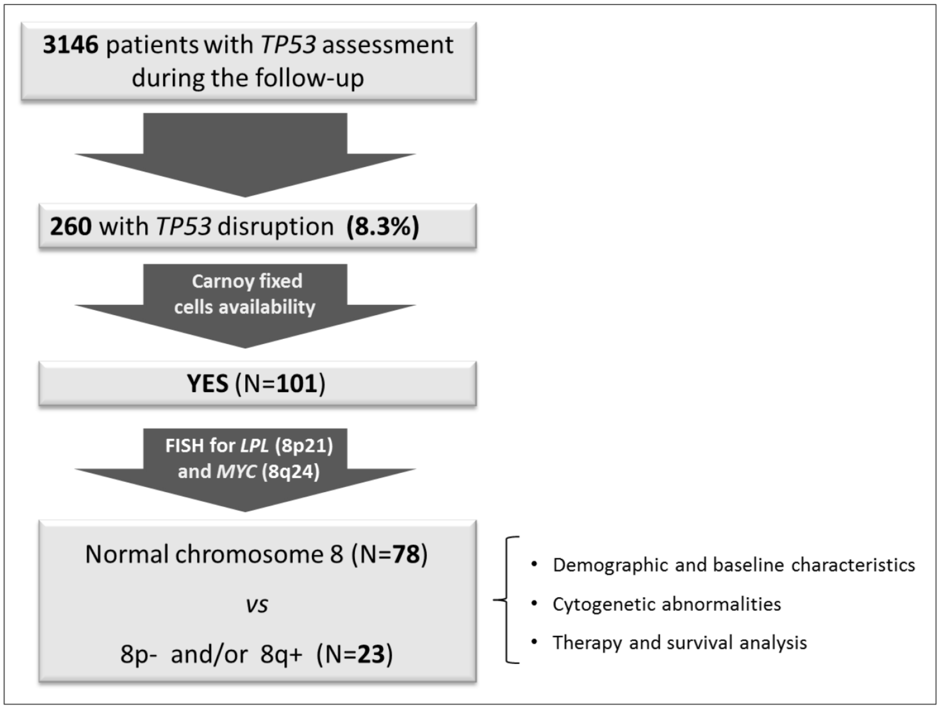

Supplementary Figure S3: Representation of the procedure used to select the study cohort.
